# Evaluation of a rapid turn-over, fully-automated ADAMTS13 activity assay: a method comparison study

**DOI:** 10.1007/s11239-020-02086-8

**Published:** 2020-03-26

**Authors:** Jan Stratmann, Josephine-Nana Ward, Wolfgang Miesbach

**Affiliations:** 1 Department of Hemostaseology and Hemophilia Center, University Hospital Frankfurt, Theodor Stern Kai 7, 60596 Frankfurt, Germany; 2 Department of Internal Medicine, Institute of Transfusion Medicine, University Hospital Frankfurt, Sandhofstraße 1, 60528 Frankfurt, Germany; 3 Department of Hematology and Oncology, Johann Wolfgang Goethe University of Frankfurt, Theodor Stern Kai 7, 60596 Frankfurt am Main, Germany

**Keywords:** Thrombotic thrombocytopenic purpura, ADAMTS13, Diagnosis, Management, Test assay

## Abstract

Thrombotic thrombocytopenic purpura (TTP) is a life-threatening thrombotic microangiopathy caused by severely reduced activity of the von-Willebrand factor-cleaving protease ADAMTS13, mainly caused by anti-ADAMTS-13 antibodies. Although several test systems for ADAMTS13 measurement exist, long turn-around times hamper the usability in daily practice. We performed a method comparison study for two commercially available ADAMTS13 assays and evaluated the agreement between the fully-automated rapid turn-over HemosIL AcuStar ADAMTS13 Activity assay and the manually performed TECHNOZYM ADAMTS-13 Activity assay. Twenty-four paired test samples derived from 10 consecutively recruited patients (n = 8, acquired TTP; n = 1, atypical hemolytic uremic syndrome; n = 1, control), of which nine test samples were collected in case of clinically apparent TTP and 13 samples were collected from TTP patients in clinical remission were included. Overall correlation between the TECHNOZYM and AcuStar assay was good with a Pearson R of 0.93 (p < 0.001). Agreement between the assays assessed with the Passing–Bablok analysis showed high agreement with an Intercept of  − 2.56 (95% confidence interval [CI], − 5.07 to  − 0.86) and Slope of 1.04 (95% CI 0.84–1.17). The absolute mean bias was 2.54% (standard difference [SD], 6.38%; 95% CI to 10.0–15.05%). Intra-method reliability was high with an absolute mean bias of − 0.13% (SD 3.21%; 95% CI to 6.42–6.16%). The observer agreement for categorial thresholds (> or < 10% ADAMTS3 activity) was kappa = 0.82 (95% CI 0.59–1.0). Conclusively, overall agreement between the testing methods was sufficient and we support previously published data suggesting the AcuStar assay being a valuable and accurate tool for ADAMTS13 activity testing and TTP diagnostics.

## Highlights

- Thrombotic thrombocytopenic purpura is a life-threatening disease and making the correct diagnosis is crucial for successful treatment
- Long turn-around times of ADAMTS13 assays hamper the usability in daily practice
- The new rapid turn-over HemosIL AcuStar ADAMTS13 Activity assay shows high agreement with a standard-of-care TECHNOZYME assay
- Patients with clinical active TTP are successfully identified with the AcuStar assay
- Performance in fresh plasma samples still needs confirmation in additional studies

Thrombotic thrombocytopenic purpura (TTP) is a life-threatening thrombotic microangiopathy caused by severely reduced activity of the von-Willebrand factor-cleaving protease ADAMTS13 (a disintegrin and metalloprotease with thrombospondin motifs 13), mainly caused by anti-ADAMTS-13 antibodies (acquired TTP). It is characterized by hemolytic anemia and small-vessel clot formations leading to consumptive thrombocytopenia and end-organ damage, frequently affecting the kidneys and the central nervous system. The natural course of disease is almost always fatal, however, with appropriate treatment, survival rates exceed 90% underscoring the need for rapidly making the correct diagnosis. Complementing anamnestic and clinical findings, measurement of ADAMTS13 activity is pivotal for confirming suspected TTP and a threshold below 10% activity is considered to be affirmative [1].

Although several test systems for ADAMTS13 measurement exist [2], long turn-around times often enforce therapy initiation with plasma-exchange based solely on clinical assessment, potentially misdistributing human and economic resources and most importantly, leaving the patient at risk for inadequate treatment for their condition [3, 4]. Therefore, a rapid test result for ADAMTS13 activity to confirm or dismiss the suspected diagnosis of TTP has the potential to improve early treatment decisions in these usually critically ill patients.

We performed a method comparison study for two commercially available ADAMTS13 assays and evaluated the agreement between the fully-automated HemosIL AcuStar ADAMTS13 Activity assay (Instrumentation Laboratory, Werfen Company, Bedford, Massachusetts, United States) with an assay length of 35 min (assay range of 0.2–150.0%; IU/ml ADAMTS13 activity) and the manually performed TECHNOZYM ADAMTS-13 Activity assay (Technoclone GmBH, Vienna, Austria; assay range of 0.01 to app. 150% IU/ml ADAMTS13 activity) with an assay length of approximately 3.5 h (2.2 h cumulative for incubation and measurement in addition to 6 washing and mixing steps of variable length [5]) according to the package insert information. Blood specimen were derived from patients who were treated at the Comprehensive Care Hemophilia Center of the University Clinic Frankfurt am Main, Germany between 10/2016 and 08/2019.

Blood samples were collected in 10 ml sodium-citrate buffer solution (S-Monovette®, Sarstedt, Nümbrecht, Germany) yielding a final buffer:blood ratio of 1:9 according to the manufacturer´s instructions. Plasma was separated and equally aliquoted samples were stored at – 70 °C until further (and simultaneous) testing on the stated assays according to the manufacturer´s instructions.

Differences between testing methods were evaluated using the paired t-test. Correlation between methods was assessed using the Pearson R coefficient and agreement was analysed using the Passing-Bablok regression test and the Bland Altman analysis. Additionally, we explored the re-test reliability of the AcuStar assay by performing test doublets and comparing fresh (immediate testing) to frozen plasma samples (rethawed after 1–3 days). All variables collected were processed using Prism, Version 6 (GraphPad Software, USA) and Analyse-it Method Validation Edition, Version 5.2 (Analyse-it Software, USA). Approval from the responsible institutional review board was obtained before data acquisition. Written informed consent was obtained from all individual participants included in this study. All procedures were in accordance with the ethical standards of the institutional research committee and with the 1964 Helsinki declaration.

Twenty-four paired test samples derived from 10 consecutively recruited patients (n = 8, acquired TTP; n = 1, atypical hemolytic uremic syndrome; n = 1, normal control), of which nine test samples were collected in case of clinically apparent TTP (acute thrombocytopenia, schistocytes on peripheral smear) and 13 samples were collected from TTP patients in clinical remission were included.

**Fig. 1:**
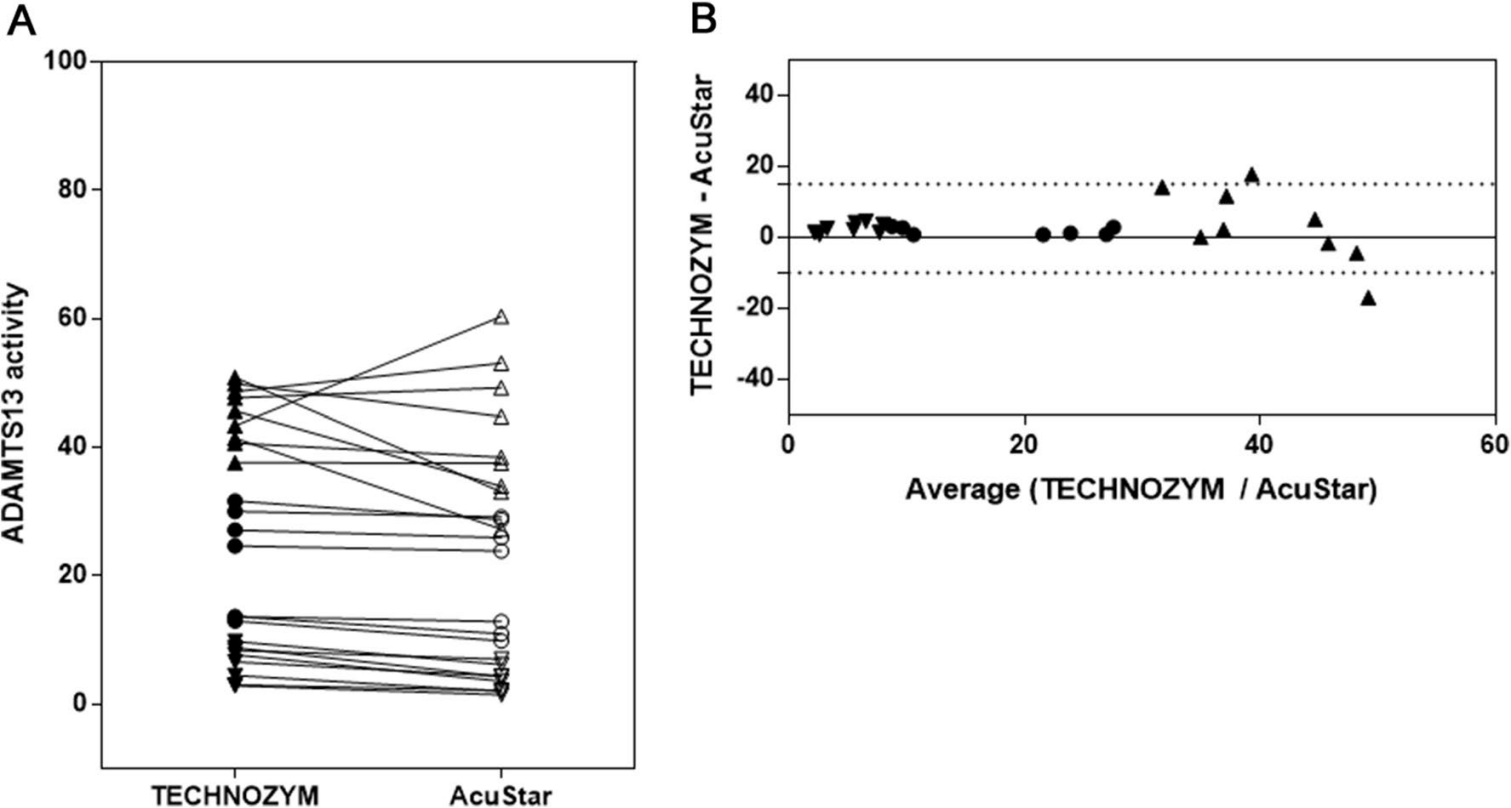
**a** Single value plot and **b** Bland Altmann plot for method comparison: TECHNOZYM vs AcuStar; triangle with tip pointing downwards shows activity values < 10% ADAMTS13, circles mark activity values between 10–30%, triangle with tip pointing upwards shows activity values > 30%

Descriptive and correlation analyses are shown in Table 1. Overall correlation between the TECHNOZYM and AcuStar assay was good with a Pearson R of 0.93 (p < 0.001) without significant differences in the paired two-sided T test (p = 0.06). Agreement between the assays as assessed with the Passing-Bablok analysis showed high agreement with an Intercept of –2.56 (95% confidence interval [CI], − 5.07 to − 0.86) and Slope of 1.04 (95% CI 0.84–1.17). The absolute mean bias reported from the Bland Altmann analysis was 2.54% (standard difference [SD], 6.38%; 95% CI − 10.0–15.05%) as shown in Fig. 1. Inter-method agreement was higher among lower ADAMTS13 values below a threshold of 30% activity, whereas there was less agreement in values above the stated activity level. This is also underscored by subgroup analyses presented in Table 1 showing less correlation and significant differences between the TECHNOZYM and AcuStar in ADAMTS13 activity values above a threshold of 30%. The observer agreement for categorial thresholds (> or < 10% ADAMTS3 activity) was kappa = 0.82 (95% CI 0.59–1.0).

**Table 1:** Descriptive statistics of ADAMTS13 method comparison

Retest reliability (only AcuStar)	AcuStar	AcuStar	Pearson R p value	t test p value
Number of values	25	25		
Median (range)	23.5 (1.4–83.9)	23.1 (1.5–96.8)	0.99; p < 0.0001	0.83
5%, 95% Percentile	1.6, 75.5	1.7, 85.6		

Material preparation (only AcuStar)	Fresh (AcuStar)	Frozen (AcuStar)	Pearson R p value	t test p value
Number of values	6	6		
Median (range)	97.9 (77.1–120.1)	85.7 (69.3–113.3)	0.81; p = 0.05	0.08
5%, 95% Percentile	77.1, 120.1	69.3, 113.3		

Method Comparison	TECHNOZYM	AcuStar	Pearson R p value	t test p value
Number of values	24	24		
Median (range)	23,25 (2.9–48.2)	22.25 (1.5–57.7)	0.929; p < 0.0001	0.06
5%, 95% Percentile	2.9, 47.9	1.6, 55.9		

ADAMTS13 Subgroups	TECHNOZYM	AcuStar		
0–10% (median, range)	7.2 (2.9–9.8)	4.0 (1.5–7.0)	0.87; < 0.001	0.001
10–30% (median, range)	22.0 (10.3–29.0)	21.2 (7.2–26.5)	0.99; < 0.001	0.005
> 30% (median, range)	43.0 (35.0–48.2)	35.8 (24.6–57.7)	0.21; 0.57	0.41
caTTP (median, range)	7.7 (2.9–11.0)	4.3 (1.5–10.2)	0.88; < 0.001	0.001
No TTP	38.0 (10.3–48.2)	30.4 (7.2–57.7)	0.82; < 0.001	0.23

With regards to the intra-method reliability (AcuStar only) assessed with the Bland-Altmann method, we encountered a high agreement in previously frozen plasma (n = 25) samples with an absolute mean bias of − 0.13% (SD 3.21%; 95% CI − 6.42–6.16%). There was less agreement comparing fresh to frozen samples (n = 6) with regards to intra-method reliability (AcuStar only) with an absolute mean bias of 9.23% (SD 10.28%; 95% CI − 10.92–29.37%; Bland Altman) and systematically lower values in previously frozen plasma specimen. Of note, these six samples were not considered for the inter-method comparison.

TTP often presents with rapid and severe disease progression and treatment is often initiated upon clinical information before accurate diagnosis. Thus, valid ADAMTS13 test systems with short turn-around time would help to overcome diagnostic hurdles in this life-threatening disease. We therefore tested the accordance between two commercially available test systems, our standard of care TECHNOZYM and the novel AcuStar ADAMTS13 activity assay. Overall agreement between the testing methods was good and excellent in ADAMTS13 values below 30%. Above 30%, accordance between the testing methods was less pronounced, however, we did not see a systematic deviation. Two reported studies [5, 6] have already tested the agreement between the stated test assays and found either an average overestimation by the AcuStar assay observed in the intermediate-low ADAMTS13 activity range (mean bias 2.9% [95% CI 1.8–7.7%]) [5], whereas Favresse et al. reported lower values on average with the AcuStar assay (mean bias − 4.8% [95% CI  − 9.83–0.27%]) [6]. Our results show a trend for underestimation by the AcuStar in the intermediate-low activity range but more non-systematical dispersion in higher ADAMTS13 activity values, supporting the data provided by Favresse et al. Nevertheless, all published studies reported low absolute mean biases between the assays, and of utmost clinical importance, the biases for classifying samples with < 10% ADAMTS13 activity was near zero across all studies including ours.

We are the first to provide evidence for high intra-method reliability regarding the AcuStar assay in terms of frozen as well as comparing fresh with frozen samples. Of interest, we saw systematically higher values for fresh plasma specimen on average compared to previously frozen samples. We fully acknowledge, that our limited sample size does not allow to draw conclusions for the clinical impact of using fresh or frozen plasma samples. Still, it is noteworthy, that due to limited experience, available evidence from our and previous studies should be transferred with caution when dealing with fresh plasma specimen. Further data is needed to test the accuracy in this very setting.

Conclusively, we support previously published data suggesting the AcuStar assay being a valuable and accurate tool for ADAMTS13 activity testing and TTP diagnostics.
